# Indian Hedgehog signaling pathway members are associated with magnetic resonance imaging manifestations and pathological scores in lumbar facet joint osteoarthritis

**DOI:** 10.1038/srep10290

**Published:** 2015-05-20

**Authors:** Feng Shuang, Ying Zhou, Shu-Xun Hou, Jia-Liang Zhu, Yan Liu, Chun-Li Zhang, Jia-Guang Tang

**Affiliations:** 1 Department of Orthopedics, The First Affiliated Hospital of General Hospital of Chinese PLA, Beijing 100048, China; 2 Department of Orthopedics, The 94th Hospital of Chinese PLA, Nanchang 330002, China

## Abstract

Indian Hedgehog (HH) has been shown to be involved in osteoarthritis (OA) in articular joints, where there is evidence that Indian HH blockade could ameliorate OA. It seems to play a prominent role in development of the intervertebral disc (IVD) and in postnatal maintenance. There is little work on IHH in the IVD. Hence the aim of the current study was to investigate the role of Indian Hedgehog in the pathology of facet joint (FJ) OA. 24 patients diagnosed with lumbar intervertebral disk herniation or degenerative spinal stenosis were included. Preoperative magnetic resonance imaging (MRI) and Osteoarthritis Research Society International (OARSI) histopathology grading system was correlated to the mRNA levels of *GLI1*, *PTCH1*, and *HHIP* in the FJs. The Weishaupt grading and OARSI scores showed high positive correlation (r = 0.894) (*P* < 0.01). MRI Weishaupt grades showed positive correlation with *GLI1* (r = 0.491), *PTCH1* (r = 0.444), and *HHIP* (r = 0.654) mRNA levels (*P* < 0.05 in each case). OARSI scores were also positively correlated with *GLI1* (r = 0. 646), *PTCH1* (r = 0. 518), and *HHIP* (r = 0.762) mRNA levels (*P* < 0.01 in each case). Cumulatively our findings indicate that Indian HH signaling is increased in OA and is perhaps a key component in OA pathogenesis and progression.

Facet joint (FJ) osteoarthritis (OA) is an important component of degenerative changes in the lumbar spine, and is a major contributing factor to low back pain^1^. However, not all individuals with FJ OA develops back pain, and patients with back pain may be radiologically negative of FJ OA^2^,^3^,^4^. Early detection of FJ OA before the onset of back pain-associated symptoms may help to prevent the disease progression. At present, FJ OA is diagnosed using computed tomography (CT) or magnetic resonance imaging (MRI). Due to the poorly understood mechanisms of FJ OA, there is no available marker for the early detection of this condition.

Indian Hedgehog (Hh), sonic Hh, and desert Hh constitute the Hh family, which plays critical role in growth, patterning, and morphogenesis^5^,^6^,^7^,^8^,^9^. Indian Hh has been found to play roles in osteoarthritis^10^,^11^,^12^,^13^,^14^. It has been shown that during endochondral bone growth, Indian Hh is mainly produced and secreted by pre-hypertrophic chondrocytes and regulates chondrocyte hypertrophic differentiation. Patched1 (*PTCH1*) and Smoothened (*SMO*) are two transmembrane receptors that respond to Indian Hh signaling. When Indian Hh is not present, Patched1 inhibits Smoothened, and represses the downstream gene expression by suppressing the Gli zinc finger transcription factors (*GLI1*, *GLI2*, and *GLI3*)^15^,^16^. When Indian Hh is present, it binds to Patched and releases Smoothened, activating the Hh signaling pathway and allowing the active Gli transcription factors to enter the nucleus and enhance the transcription level of downstream targets^17^. Gli1 mainly functions as a transcription factor, and increased Gli1 level indicates activation of the Hh signaling pathway^18^,^19^. The vertebrate cell surface protein Hh-interacting protein (*HHIP*) have also been shown to bind vertebrate Hh proteins and negatively modulate Hh signaling^20^.

In this study, we examined the pathological changes in the FJ OA in humans and analyzed the expression levels of Hh signaling-associated *PTCH1, GLI1,* and *HHIP*. The prospective relationship between the pathology of FJ OA and Hh signaling is discussed.

## Material and Methods

### Ethical Statement

All experimental protocol were approved by the Ethical Committee of The First Affiliated Hospital of Chinese PLA General Hospital. Written informed consent was obtained from all enrolled patients. All the subsequent research analyses were carried out in accordance with the approved guidelines.

### Patients

This study included 24 consecutive patients (mean age of 61.7 (range 42–75 years)), including 15 males and 9 females, diagnosed with lumbar intervertebral disk herniation or degenerative spinal stenosis. These patients were treated with FJ resection *en bloc* at our hospital between October 2012 and April 2013.

### MRI

Preoperative T2-weighted MRI images of the diseased FJs were obtained and independently evaluated by two experts in this field. Discrepancy between the two evaluators were solved by discussion. The morphological MRI grading system of Weishaupt *et al.* was used to evaluate the FJs^21^,^22^. This grading system utilizes joint space width, osteophytes, facet hypertrophy, bone erosions and subchondral cysts to assign grades 0 to III, where 0 and III represents normal and the most severe changes, respectively.

### Pathological analysis

The diseased FJs were resected *en bloc* and dissected into two parts through a plane perpendicular to the joint surface. For pathological analysis, half of the FJs was fixed in 4% paraformaldehyde for 48 hours, decalcified, and embedded in paraffin. The other half of the FJs were immediately put into liquid nitrogen and used for quantitative real time polymerase chain reaction (qRT-PCR). The block was cut into 4-μm sections on the coronal plane. The sections were stained with toluidine blue and hematoxylin-eosin (HE), respectively. Osteoarthritis Research Society International (OARSI) histopathology grading system was used to evaluate the sections^23^,^24^. In this system, the grade of damage from 0 to 6 is defined as the depth of progression of OA into the cartilage and the stage of damage is defined as the horizontal extent of cartilage involvement from 0 to 4. The final score is the combined value of grade and stage (score range 0–24).

### qRT-PCR

Total RNA from the facet joints (inclusive of both the cartilage and subchondral bone) was extracted using the Trizol method (Invitrogen, US). The RNA was reversely transcribed into cDNA using random hexamers. The cDNAs were subsequently used to template qRT-PCR reactions using KAPA SYBR® FAST Universal 2X qPCR Master Mix (KAPA BIOSYSTEMS, Wilmington, MA, USA) using primers mentioned in Table 1 and following manufacturer recommended reaction conditions. The data were analyzed with 2^−ΔΔCt^ method and normalized to *ACTB* expression.

### Statistical analysis

Continuous data were expressed as mean ± SD. Correlation analysis was performed using Spearman rank coefficient with SPSS 17.0 (SPSS, US). A *P*-value less than 0.05 was considered statistically significant.

## Results

### MRI image grades

The Weishaupt grades included grade 0 in 5 cases, showing normal FJ space of 2–4 mm; grade I in 8 cases, showing narrowed FJ space of <2 mm, small osteophytes, and mild articular hypertrophy; grade II in 7 cases, showing narrowed FJ space, moderate osteophytes and articular hypertrophy, mild subchondral erosion; grade III in 4 cases, showing narrowed FJ space, large osteophytes, severe articular hypertrophy, subchondral erosion and cysts (Table 2).

#### Pathological examination

**Figure 1:**
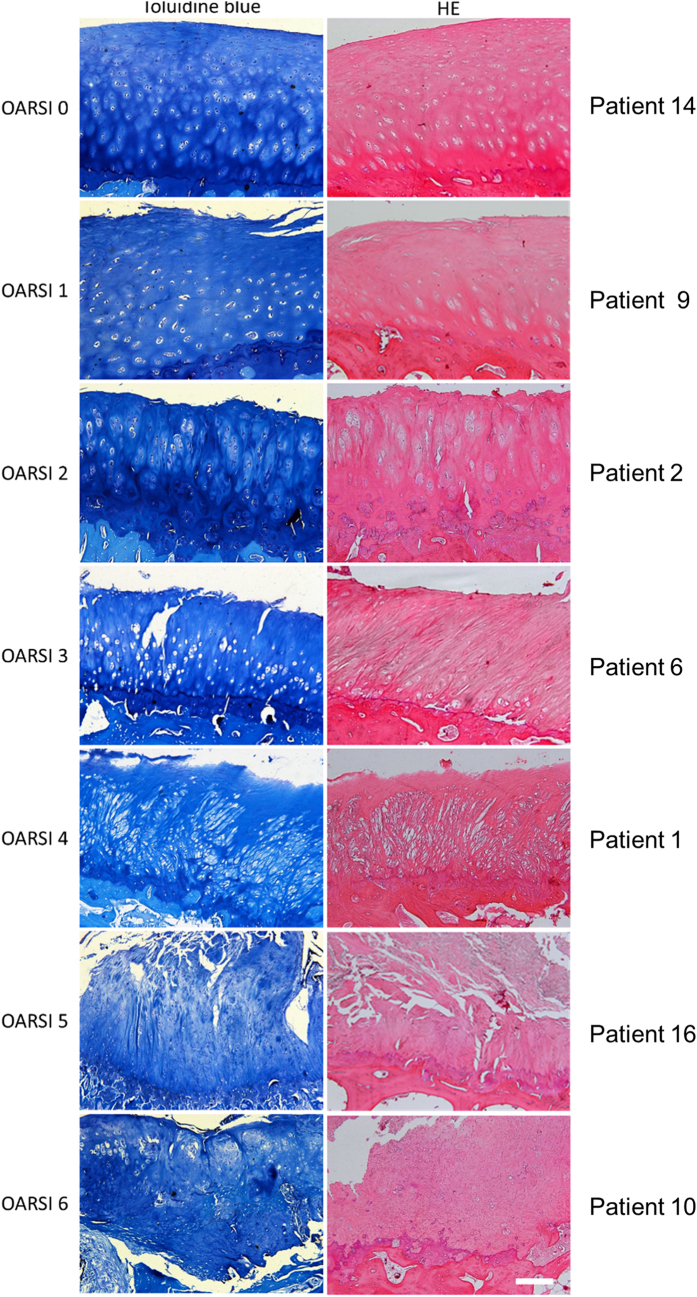
Toluidine blue and HE images of the FJs showing OARSI grades 0–6.

The toluidine blue and HE staining results were scored with OARSI grading system (Table 2). Typical pathological images and corresponding patient information are shown in Fig. 1. Grade 0 showed intact articular surface, clear layer structures, and normal matrix. Grade 1 showed fibrosis in the articular surface and chondrocyte hypertrophy (relative increase of chondrocyte cytoplasm compared to other chondrocytes in the histologic cartilage layer). Grade 2 showed matrix discontinuity and chondrocyte hypertrophy. Grade 3 showed cracks in the matrix and chondrocyte necrosis. Grade 4 showed matrix loss and cyst formation. Grade 5 showed exposure of the subchondral bone and fibrocartilage repair. Grade 6 showed bone reconstruction and microfracture in the fibrocartilage.

#### Correlation analysis

**Figure 2:**
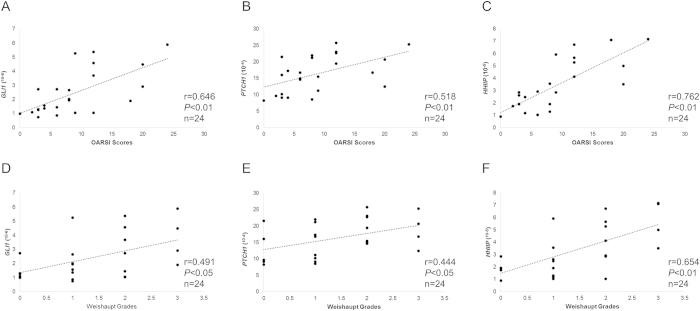
Correlation analysis between OARSI scores (**A–C**) and Weishaupt grade (**D–F**) and mRNA levels of *GLI1*, *PTCH1*, and *HHIP*.

**Figure 3:**
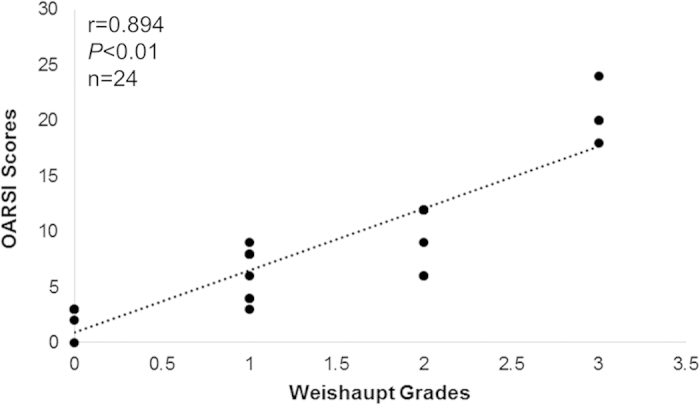
Correlation analysis between OARSI scores and Weishaupt grade.

OARSI scores were positively correlated with GLI1 (r = 0. 646), PTCH1 (r = 0. 518), and HHIP (r = 0.762) mRNA expression levels (*P* < 0.01 in each case) (Fig. 2A–C). MRI Weishaupt grades were positively correlated with GLI1 (r = 0.491), PTCH1 (r = 0.444), and HHIP (r = 0.654) mRNA expression levels (*P* < 0.05) (Fig. 2D–F). Furthermore, pathological OARSI scores were positively correlated with MRI Weishaupt grades (r = 0.894, *P* < 0.01) (Fig. 3).

## Discussion

In this study, we collected FJs with OA from 24 patients and examined the mRNA levels of Hh signaling-associated genes. It was found that the mRNA levels of GLI1, PTCH1, and HHIP are positively associated with the MRI image and pathological scores of the FJs, suggesting a role of the Indian Hh pathway in the development of FJ OA.

It has been shown that ablation of Indian Hh from postnatal chondrocytes leads to closure of the growth plate, loss of trabecular bone, and defective skeletal growth^25^,^26^, suggesting that Indian Hh plays important roles in postnatal growth. In addition, Indian Hh is induced after femoral fracture in adults, suggesting that Indian Hh might be involved in the repair after bone injury^27^,^28^, Indian Hh plays a critical role in endochondral bone formation in embryo and limb development as well as postnatal bone formation. However, its expression starts to decrease with aging under physiological conditions^29^. In a human study, Indian Hh was almost undetectable in healthy adult cartilage but it increased in early cartilage damage^30^.

In human OA cartilage and synovial fluid, Indian Hh is upregulated and is correlated with OA progression^12^. Changes in gene expression and chondrocyte morphology that are consistent with chondrocyte hypertrophy and cartilage degeneration were also found in OA cartilage. These findings provide strong evidence that Indian Hh plays a critical role in OA pathology.

In addition, genetically modified mice that have elevated Hh signaling in chondrocytes showed more severe OA phenotype, and OA-caused cartilage damage can be alleviated by inhibiting Hh signaling in mice or human cartilage explants^31^. Lin *et al.* had reported that *PTCH1*, *GLI1*, and *HHIP* were all overexpressed in human osteoarthritic samples, as well as in the articular cartilage from a mouse model of osteoarthritis. In addition, mice with aberrant activation of the Hh pathway showed cartilage degradation and an increased tendency to develop osteoarthritis. The severity of the disease correlated with the degree of Hh pathway activation. Furthermore, genetic or pharmacologic inhibition of the Hh pathway in a mouse model of osteoarthritis significantly decreased collagen X deposition, cartilage degradation and osteoarthritis severity. Remarkably, inhibition of Hh in explant cultures of human osteoarthritic cartilage samples blocked expression of key genetic markers of osteoarthritis, including the metalloproteinases *ADAMTS5* and *MMP13*, the transcription factor *RUNX2*, and *COL10A1*^31^. Conditional deletion of Indian Hh in chondrocytes can attenuate OA progression in a mouse model^14^. Given all this information and our findings it would be rational to presume that IHH signaling is increased in OA and is perhaps a key component in OA pathogenesis and progression. However, our future endeavors would be geared towards specifically testing the same.

In our study, the mRNA expression levels of three Indian Hh-associated genes, *GLI1*, *PTCH1*, and *HHIP*, were found to be positively correlated with FJ OA pathological scores. This is the first study demonstrating a role of Indian Hh in the pathology of FJ OA. Future endeavors will deal with defining the precise role and underlying molecular mechanism by which Indian Hh signaling promote FJ OA.

## Author Contributions

F.S. and S.X.H. wrote the main manuscript text, J.L.Z., Y.L. and Y.Z. collected data and carried out the experiments, C.L.Z. analyzed the data, J.G.T. designed the experiments. All author has approved the manuscript.

## Additional Information

**How to cite this article**: Shuang, F. *et al.* Indian Hedgehog signaling pathway members are associated with magnetic resonance imaging manifestations and pathological scores in lumbar facet joint osteoarthritis. *Sci. Rep.* **5**, 10290; doi: 10.1038/srep10290 (2015).

## Figures and Tables

**Table 1:** Primers used for RT-PCR.

**Primer**	**Sequence**	**Product (bp)**
*GLI1*-F	5′-GGTGGTTCACATGCGCAG-3′	170
*GLI1*-R	5′-CATTGCTGAAGGCTTTACTGC-3′	
*PTCH1*-F	5′-CCACCAAGTGATCGTGGAAG-3′	244
*PTCH1*-R	5′-GCCAGAATGCCCTTCAGTAGA-3′	
*HHIP*-F	5′-TCCGGTCACATCTTGGGATT-3′	167
*HHIP*-R	5′-GTCTGTGCAGGTTGTACCGTG-3′	
*ACTB*-F	5′-TCCCTGGAGAAGAGCTACG-3′	131
*ACTB*-R	5′-GTAGTTTCGTGGATGCCACA-3′	

**Table 2:** Pathological scores and mRNA levels of GLI1, PTCH1, and HHIP.

**Patient number**	**Weishaupt grades**	**OARSI scores**	***GLI1*** **(10**^**−6**^)	***PTCH1*****(10**^**−4**^)	***HHIIP*** **(10**^**−5**^)
1	3	18	1.893	16.736	7.104
2	1	9	5.258	11.200	5.909
3	1	8	2.014	8.564	1.909
4	2	6	1.439	14.970	2.909
5	0	3	1.262	9.103	2.844
6	2	12	1.042	25.754	6.730
7	1	6	0.859	16.726	1.026
8	0	3	1.293	16.052	1.907
9	1	4	1.563	17.253	1.167
10	3	24	5.887	25.300	7.179
11	3	20	4.482	20.690	3.502
12	2	12	5.377	19.448	5.650
13	1	4	1.357	9.137	2.462
14	0	0	0.986	8.245	0.890
15	1	3	0.748	10.172	2.610
16	3	20	2.914	12.450	4.990
17	2	9	1.056	15.470	2.844
18	0	2	1.096	9.640	1.730
19	2	6	2.7212	14.600	1.026
20	0	3	2.7199	21.550	1.907
21	2	12	3.6874	22.680	5.282
22	1	8	2.6554	21.220	3.554
23	2	12	4.5783	23.045	4.113
24	1	8	1.9477	21.954	1.292
